# Hand Grip Strength and Myocardial Oxygen Consumption Index among Coronary Artery Bypass Grafting Patients

**Published:** 2015-07

**Authors:** Siti Nur Baait Biniti Mohd Sokran, Vikram Mohan, Kamaria Kamaruddin, Mohd Daud Sulaiman, Yahya Awang, Ida Rosmini Binti Othman, Smiley Jesu Priya Victor

**Affiliations:** 1 Department of Physiotherapy, Faculty of Health Sciences, Universiti Teknologi MARA, Puncak Alam, Selangor, Malaysia;; 2 KPJ Damansara Specialist Hospital, Damansara Utama, Petaling Jaya, Selangor, Malaysia;; 3 Center for Statistical Studies, Faculty of Computer and Mathematical Sciences, Universiti Teknologi MARA, Shah Alam, Selangor, Malaysia;; 4 Department of Occupational Therapy, Faculty of Health Sciences, Universiti Teknologi MARA, Puncak Alam, Selangor, Malaysia

**Keywords:** Coronary artery disease, Hand strength, Oxygen consumption

## Abstract

**Background:**

Hand grip strength (HGS) is a reliable indicator of peripheral muscle strength. Although, numerous studies have investigated the strength of hand grip; little attention has been given to coronary artery disease (CAD) patients, exploring the relationship between HGS and myocardial oxygen consumption (MVO_2_) index. The current study aimed to evaluate the interaction between HGS and MVO_2 _index findings before and after cardiac surgery.

**Methods:**

Twenty-seven patients with CAD had HGS were assessed using handheld dynamometer. HGS for each hand were documented. MVO_2 _index was assessed using rate pressure product (RPP), which is the product of the heart rate (HR) and systolic blood pressure (SBP). Repeated measures MANOVA were carried out to estimate the interaction between both hands HGS and MVO_2_ index before and after surgery.

**Results:**

There was significant interactions (P<0.001) for both HGS dominant and non-dominant with large effect sizes (HGS dominant×MVO_2_ index: h_p_^2^=0.44; HGS dominant×RPP: h_p_^2^=0.49). This signifies that peripheral muscle strength of the upper limb (HGS dominant and non-dominant) had different effects on MVO_2_ index before and after surgery. The interaction graph shows that the increase in MVO_2_ index after surgery was significantly greater for peripheral muscle strength of the dominant hand when compared to non-dominant.

**Conclusion:**

Patients with CAD had interactions between HGS and oxygen consumption before and after surgery. Hence, HGS might be used as a predictor to assess oxygen consumption among cardiac patients.

## Introduction

Coronary artery disease (CAD) patients are unremarkably associated with various risk factors and physical limitation.^1^^,^^2^ Potential identified risk factors for both male and female are hypertension, diabetes, obesity, smoking, and physical inactivity as reported by Tan et al.^2^ It was identified that there is greater association between diabetes and stroke in South Asians and African Caribbean’s compared with Europeans in a recently published population based study.^3^ Hence, these risk factors which strikes different ethnic groups under certain circumstances, might predisposes to lower fitness level and thereby cause various physical limitations. However, these rapid changes on the accounted disease with these risk elements are inducing a serious consequence in the activity level of an individual.

One of the accounted physical limitations includes abnormal peripheral muscle functions, which are well documented among cardiac patients.^4^ Furthermore, the peripheral muscle function declines in cardiac surgical patients where they will undergo alterations in lean body mass.^5^ The alteration in lean body mass inclines to severe loss of skeletal muscle mass (SMM) in both upper and lower limbs. Loss of SMM is also associated with persistent muscle weakness, impaired cardiac function that will limit the exercise capacity as well myocardial oxygen consumption.

Peripheral muscle weakness is in turn associated with reduced muscle strength and loss of physical function.^6^ In these position, grip strength measurement is conceived to be useful for assessing the overall muscle strength and it shows a linear association with chronic diseases such as chronic obstructive airways disease, hyperlipidaemia, diabetes and ischemic heart diseases as described by Cheung et al.^7^ The strength of the hand grip may be measured using dynamometer, which is a good indicator for the measurement of peripheral muscle strength of the upper limb. Hand grip strength also shows a linear correlation with the level of impairment of cardiac function and exercises such as isometric hand grip training has been used as one of the training protocols for medicated hypertensive patients.^8^^,^^9^ However, data comparing pre- and post-operative peripheral muscle strength of the upper limb among CAD subjects is deficient.

Impaired cardiac function, leading from CAD, leads to an imbalance between oxygen supply and the demands of the heart. Imbalance in the myocardial oxygen demands in response to varied coronary circulation that can be assessed by means of myocardial oxygen consumption (MVO_2_).^1^ Studies established a linear relationship between coronary blood flow and MVO_2_.^10^ Indirect measurement of MVO_2 _can be performed by means of rate pressure product (RPP) or MVO_2 _index.^10^ Furthermore; MVO_2 _is a strong predictor of exercise capacity, which in turn can predict peripheral muscle function.

Although, numerous studies have investigated the strength of hand grip (HGS), little attention has been given to CAD subjects exploring the relationship between HGS and MVO_2_. However, the interaction of MVO_2 _in relation to coronary blood flow and hand grip strength among CAD subjects has not been studied. In addition, these important measurements are not routinely used in all rehabilitation setup. Hence, the potential implications of the study might contribute to the welfare of individual cardiac patients who are participating in rehabilitation. Therefore, the preliminary study was aimed to assess the interaction between peripheral muscle function of the upper limb and MVO_2 _index among CAD patients pre- and post-cardiac surgery.

## Materials and Methods

*Study Design*

In this single-centered prospective study, all patients underwent open-heart surgery at a private hospital in Malaysia between June and September 2012. Following Institutional and Hospital Ethical Committee approval, a sample size of 29 patients was estimated initially with correlation H1=0.5 and 80% power by G*Power 3 software (Faul et al.).^11^ Two patients were excluded from this study, as they did not meet the criteria as set by the study protocol. Hence, 27 patients were included after obtaining informed consent. The inclusion criteria in both female and male were: diagnosed as CAD, aged ≥19 years old, left ventricular ejection fraction (LVEF) ≤55% and New York Heart Association criteria (NYHA) II or III.^12^^,^^13^Patients with peripheral or vascular problem of the upper limb, abnormal body geometry such as amputation of the upper limbs and unstable vital sign were excluded from the study. Demographic data were obtained through interview technique, which included occupation, smoking habit, history of musculoskeletal or neurological problem, family history of CAD, and their physical activity level. Anthropometric measurement such as height, weight, and body mass index (BMI) were calculated using wall mounted stadiometer and weight scale (SECA Mod 220, SECA GMBH & Co. Germany).

*Rate Pressure Product (RPP) Measurement*

RPP was calculated by the product of systolic blood pressure (SBP) and heart rate (HR) such that RPP=SBP×HR/1,000.^14^ Heart rate measurement was carried out using radial pulse and the measurement of blood pressure was carried out using sphygmomanometer.

*Hand Grip Strength Measurement*

Hand grip strength measurement was executed using Jamar Hand Dynamometer (Sammons & Preston Inc., Serial No: 30906194) for both sides, based on the American Society of Hand Therapist (ASHT) recommendation. Initially the patients were seated in a comfortable position with elbow flexed at 90º, shoulder adducted, forearm in a neutral position and wrist in 0-30º extension.^15^ The dynamometer was set at the second handle position for all patients. Then, the patients were instructed to squeeze the handle for three seconds maximally and asked to avoid undue pain. The mean of three measurements for each hand was documented. Then, on the 7^th^ post-operative day, the HGS measurements and MVO_2 _index were calculated again using the same procedure.^16^

*Statistical Analysis*

Continuous variable are shown as mean and standard deviation and categorical variables as absolute values and percentages. Analysis was performed using MANOVA. SPSS version 18 (SPSS Inc., Chicago, IL, USA) was used to perform the statistical analysis. P<0.05 was considered statistically significant. 

## Results

*Profile of Patients*

All patients underwent coronary artery bypass grafting (CABG) surgery with heart-lung machine and a minimum of three vessels were involved in surgery. The commonest vessels involved were the right coronary artery, left anterior descending artery, and left circumflex artery. The vessels harvested for grafting were from the internal mammary artery or from saphenous vein. Twenty-seven patients who were involved in this study comprised of 23 males (85.2%) and 4 females (14.8%) aged between 19 years to 74 years. The mean age of male and female patients was 51.7±10.2 years and 54.5±13.7 years, respectively. The majority of patients (96.3%) were married while only one was single. More than half of the patients (55.6%) were Malays while the other 44.4% comprised of Chinese (18.5%) and Indian (25.9%). About 88.8% of the patients had tertiary (44.4%) and secondary (44.4%) education as their highest education level while the other three patients only received primary education. The BMI of the patients ranged between 17.7 to 37.3 with means and standard deviation of 27.5±4.36 with almost 60% of them were smokers. 

A repeated measures MANOVA was conducted to evaluate the interaction between peripheral muscle strength of the upper limb (HGS dominant and HGS non-dominant) and MVO_2_ index before and after cardiac surgery. Multivariate assumptions of normality and a test of sphericity assumption were met before the repeated measures MANOVA procedures were conducted. The effect size was reported as partial eta-squared (partial h_p_^2^), and was interpreted as 0.01-0.05: small effect; 0.06-0.13: moderate effect; and anything larger than 0.14: large effect.

The descriptive statistics in table 1 revealed that the HGS measurement before surgery were higher for both dominant (M_pre_=28.7 vs. M_post_=27.0) and non-dominant (M_pre_=26.0 vs. M_post_=24.0). Table 2 indicates that the main effects for both HGS dominant and HGS non-dominant for pre- and post-cardiac surgery were significant (P<0.99) with large effects (h_p_^2^=0.95). Similarly, there were also significant main effects for MVO_2_ index (P=0.99) with the measurement after surgery showed higher readings (M_pre_=10110 vs. M_post_=10228). The effect sizes for MVO_2_ index were significantly large (h_p_^ 2 ^=0.35) but lower than HGS.

**Table 1:** Descriptive statistics for main effects (HGS Dominant, HGS Non Dominant and MVO_2_ index)

**Main Effect**	**Mean**	**Standard deviation**
HGS Dominant		
Pre	28.7	4.42
Post	27.0	4.55
HGS Non Dominant		
Pre	26.0	3.71
Post	24.0	3.73
MVO_2 _Index		
Pre	10110	1018
Post	10228	1861

**Table 2:** Repeated measures MANOVA for HGS Dominant, HGS Non Dominant and MVO_2_ index

	**F (1,26)**	**p**	** η_p_^2^**
Main effect			
HGS Dominant	503	<0.001	0.95
MVO_2 _Index	13.7	0.001	0.35
Interaction effect			
HGS Dominant X MVO_2 _Index	20.7	<0.001	0.44
Main effect			
HGS Non Dominant	471	<0.001	0.95
MVO_2 _Index	14.9	0.001	0.36
Interaction effect			
HGS Non Dominant X MVO_2 _Index	25.1	<0.001	0.49

**Figure 1:**
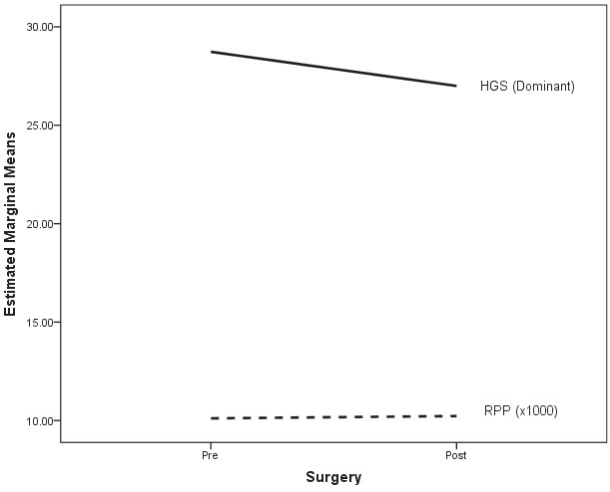
Dominant side hand grip strength (HGS) and myocardial oxygen consumption index.

**Figure 2:**
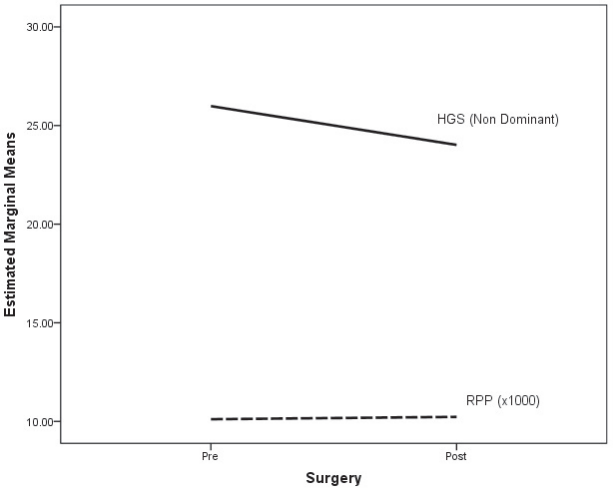
Non-dominant side hand grip strength (HGS) and myocardial oxygen consumption index.

Regarding the interaction effects, both revealed significant results (P<0.99) with large effect sizes (HGS dominant×MVO_2_ index: h_p_^ 2 ^=0.44; HGS non-dominant×MVO_2_ index: h_p_^ 2 ^=0.49). This indicates that peripheral muscle strength of the upper limb (HGS dominant and HGS non-dominant) had different effects on MVO_2_ index before and after cardiac surgery. Figure 1, depicts the interaction graph which shows that the increase in MVO_2_ index after surgery with reduced HGS for dominant hand. Figure 2, describes the interaction graph which shows that the increase in MVO_2_ index after surgery with reduced HGS in non-dominant hand.

## Discussion

The general results of this study showed that there were significant interaction effects between both HGS (dominant and non-dominant) and MVO_2_ index prior to and after surgery. This establishes that following revascularization surgery, the non-invasive measure of using MVO_2_ improved substantially which demonstrates the amount of pumping capacity amended significantly, whereas HGS measurement decreased relatively following the surgery. To the best of our knowledge, this is the first study to show the difference between the HGS of CAD patients before and after surgery and the interaction between HGS and MVO2 index.

Postoperative muscle weakness is a common problem among CAD patients as well as after open-heart surgery. In this regard, we witnessed a reduction in HGS (dominant and non-dominant) among CAD patients. The same trend was followed in our study following open-heart surgery, which further deteriorated the HGS in non-dominant hand when compared with the dominant hand. There were similarities between the results expressed by the present study and those described by van Venrooij et al. in which they established that postoperative arm muscle mass loss following CABG patients than in patients who underwent heart valve surgery.^5^ The possible explanation to the consequence is probably due to the decreased activity of local muscular oxidative enzymes and smaller muscle cross-sectional area.^4^

The results of the present study, in line with MVO_2 _index, were found to be increased following surgery and were associated with decrease in HGS. An increase in MVO_2 _index suggests improvement in blood supply to the heart muscle, whereas a decrease in HGS following surgery suggests diluted peripheral muscle strength following surgery.^3^

It is encouraging to compare this result with that reported by Nagpal et al. who found that percentage increase in MVO_2 _index was significantly more in postmenopausal women following exercise.^14^ These findings further support the idea of the present work in which it was determined that MVO_2 _index improved following surgical intervention as a mode of treatment. There are several possible explanations for these results; initially it can be argued physiologically that an increase in MVO_2 _index could be due to a better coronary perfusion in which there will be a gain in myocardial blood flow and myocardial blood volume following surgery.^17^ The observed increase in MVO_2 _index could be attributed to an increase in the left ventricular function following CABG surgery.

However, the results of this present study need to be interpreted with care because of several study limitations. First, there was an insufficient sample size since two patients were withdrawn due to predetermined criteria set by the study protocol and gender inequality. However, this preliminary study has created research interest in the field of rehabilitation. Second, examination of hand grip strength and MVO_2 _index is exercised only on the 7^th^ day, as this measurement can be carried out after a month to know the carry over effect following surgical management. Hence, the results cannot be generalized. Third, the age ranges of the patients were between 19 to 54 years old. Similarly, the results of the study may not be appropriate in such preliminary stage for clinical decision-making. However, as a part of an assessment tool or rehabilitation measures, HGS can be considered as one of the measures in cardiac rehabilitation protocols since it is a predictor of multimorbidity as reported by Cheung et al.^7^Therefore, more research on this topic needs to be undertaken with larger samples and with narrower age range before the association between these variables is more clearly understood.

## Conclusion

The findings from this study demonstrated that HGS had different effects on MVO_2 _index prior to and after CABG surgery. Hence, HGS might be used as a predictor to assess oxygen consumption among cardiac subjects.
